# Raptor is Phosphorylated by cdc2 during Mitosis

**DOI:** 10.1371/journal.pone.0009197

**Published:** 2010-02-12

**Authors:** Dana M. Gwinn, John M. Asara, Reuben J. Shaw

**Affiliations:** 1 Molecular and Cell Biology Laboratory, Dulbecco Center for Cancer Research, La Jolla, California, United States of America; 2 Howard Hughes Medical Institute, Salk Institute for Biological Studies, La Jolla, California, United States of America; 3 Division of Signal Transduction, Beth Israel Deaconess Medical Center, Boston, Massachusetts, United States of America; 4 Department of Medicine, Harvard Medical School, Boston, Massachusetts, United States of America; University of Washington, United States of America

## Abstract

**Background:**

The appropriate control of mitotic entry and exit is reliant on a series of interlocking signaling events that coordinately drive the biological processes required for accurate cell division. Overlaid onto these signals that promote orchestrated cell division are checkpoints that ensure appropriate mitotic spindle formation, a lack of DNA damage, kinetochore attachment, and that each daughter cell has the appropriate complement of DNA. We recently discovered that AMP-activated protein kinase (AMPK) modulates the G2/M phase of cell cycle progression in part through its suppression of mammalian target of rapamycin (mTOR) signaling. AMPK directly phosphorylates the critical mTOR binding partner raptor inhibiting mTORC1 (mTOR-raptor rapamycin sensitive mTOR kinase complex 1). As mTOR has been previously tied to mitotic control, we examined further how raptor may contribute to this process.

**Methodology/Principal Findings:**

We have discovered that raptor becomes highly phosphorylated in cells in mitosis. Utilizing tandem mass spectrometry, we identified a number of novel phosphorylation sites in raptor, and using phospho-specific antibodies demonstrated that raptor becomes phosphorylated on phospho-serine/threonine-proline sites in mitosis. A combination of site-directed mutagenesis in a tagged raptor cDNA and analysis with a series of new phospho-specific antibodies generated against different sites in raptor revealed that Serine 696 and Threonine 706 represent two key sites in raptor phosphorylated in mitosis. We demonstrate that the mitotic cyclin-dependent kinase cdc2/CDK1 is the kinase responsible for phosphorylating these sites, and its mitotic partner Cyclin B efficiently coimmunoprecipitates with raptor in mitotic cells.

**Conclusions/Significance:**

This study demonstrates that the key mTOR binding partner raptor is directly phosphorylated during mitosis by cdc2. This reinforces previous studies suggesting that mTOR activity is highly regulated and important for mitotic progression, and points to a direct modulation of the mTORC1 complex during mitosis.

## Introduction

The serine/threonine protein kinase mammalian target of rapamycin (mTOR) is a key mediator of the cellular response to nutrient status through its regulation of translation, ribosome biogenesis, mitochondrial metabolism, and autophagy [1]. mTOR is present in one of two complexes within the cell: mTORC1 is defined by raptor, GbL/mLST8, and negative regulatory subunits PRAS40 and DEPTOR, whereas mTORC2 contains rictor, mSin1, and Protor as well as GbL/mLST8 and DEPTOR [2].

The best-established substrates of mTORC1 demonstrate the importance of mTOR in translational control. mTOR phosphorylates S6K1 at T389 to enhance S6K1 activity, which amongst other things phosphorylates the S6 subunit of the ribosome to promote translation. mTOR also phosphorylates 4EBP1, causing its dissociation from its binding partner eIF4E, which is then free to associate with the cap-complex to promote cap-dependent translation [3].

The activity of mTORC1 is dependent on the small Ras-like GTPase, Rheb, whose GTP-loaded state is regulated by a GTPase-accelerating protein (GAP) complex composed of the TSC1 and TSC2 tumor suppressors. Inputs from a variety of pathways converge on the TSC1/2 complex to regulate mTORC1 signaling [4]. Following growth factor stimulation, Akt, Erk and Rsk can phosphorylate and inactivate TSC2, leading to activation of mTORC1. Under conditions of low ATP, the energy-sensing kinase AMPK is activated and phosphorylates and activates TSC2, inhibiting mTORC1.

In addition to the hub of signaling at TSC2, phosphorylation of components of mTORC1 have recently been shown to have important regulatory roles in mTOR signaling [5], [6], [7], [8], [9], [10], [11]. PRAS40 is a substrate of both Akt and mTOR, where upon phosphorylation, PRAS40 dissociates from mTORC1, relieving inhibition of mTORC1 activity following growth factor stimulation. mTOR also phosphorylates the recently identified mTORC1 component DEPTOR, marking it for degradation and further alleviating inhibition of mTORC1 [2]. Raptor (regulatory associated protein of TOR) is thought to act as the key mTORC1 scaffolding protein that binds mTOR substrates via the TOR signaling (TOS) motif, facilitating their phosphorylation by mTOR. A handful of recent studies have demonstrated the importance of phosphorylation of raptor on various sites in the regulation of mTOR signaling by pro- and anti-proliferative signals. Phosphorylation by Rsk at S721 as well as by mTOR at S863 have been shown to enhance mTORC1 activity [11], whereas phosphorylation at S722 and S792 by AMPK create 14-3-3 binding sites and inhibit mTORC1 activity [10]. The exact mechanism of augmentation or inhibition of mTOR activity by raptor phosphorylation remains elusive.

We have shown previously that under energy stress conditions, fewer cells proceed into G2/M and that this cell cycle arrest is dependent on AMPK phosphorylation of raptor and inhibition of mTORC1 activity. This suggested that perhaps mTOR signaling might play a role in mitosis, as suppression of mTOR blocks entry into G2/M and inappropriate activation of mTOR signaling drives cells into G2/M. In our investigations into the regulation of mTOR signaling in mitosis, we identified several sites in raptor phosphorylated by Cdc2 that may play a role in mitotic progression.

## Materials and Methods

### Antibodies and Plasmids

Myc-raptor, AU1-mTOR, HA-GbL and Flag-PRAS40 originated in Dr. David Sabatini's Lab (MIT, Cambridge, MA) and were obtained from Addgene.org (Cambridge, MA). Ha-tagged 4ebp1 and S6K1 were obtained from Dr. John Blenis (Harvard Medical School, Boston, MA). Myc-raptor was subcloned into pENTR3C (Invitrogen), and serine to alanine point mutations were made using QuikChange II XL (Stratagene). Mutant alleles were then put into an FBneo DEST vector by LR reaction (Invitrogen). All constructs were fully sequence verified. Phospho-Plk1 T210 was from BD Pharmingen (#558400). Phospho-raptor (S696, T706, S863, S877) and Phospho-histone H3 antibodies were obtained from Millipore. Anti-raptor used for endogenous raptor immunoprecipitations was from Invitrogen (#42-4000). The 9E10 anti-myc antibody was used for immunoprecipitations (Roche). Antibodies against raptor (#2280), mTOR (#2983), GbL (#3274), PRAS40 (#2610), phospho-S6K T389 (#9234), pAurora A, B, C (T288/T232/T198) (#2914), Cyclin B1 (#4138), phospho-4EBP1 (T37/46) (#2855), phospho-4EBP1 (S65) (#9451), myc-tag (#2272), phospho-threonine-proline (#2321), and GST-tag (#2622) were from Cell Signaling Technologies.

### Cell Culture

Hela, A549 and HEK293T cells were grown in DMEM with 10% FBS at 37° with 5% CO_2_. Cells were transfected with Lipofectamine 2000 (Invitrogen) as per manufacturer's instruction for 32–36 hours. Nocodazole (1 ug/mL) (SIGMA) or taxol (1 uM) (Cell Signaling Technologies) treatment was administered for 16–18 hours prior to lysis (usually 16 h post-transfection). Replacement of endogenous raptor with myc-tagged raptor was achieved by infecting Hela cells with a retrovirus expressing myc-wt or myc-S696/T706/S711AAA raptor in the FBneo vector, and selection in neomycin. These stables were subsequently infected with a lentivirus expressing a short-hairpin RNA that targets the 3′ UTR of human raptor in the pLKO vector and selected in puromycin and distributed by Addgene (<http://www.addgene.org/pgvec1?f=c&identifier=1858&atqx=raptor&cmd=findpl>). A549 cells were synchronized in G1/S by double thymidine block as follows: 2 mM thymidine was added to the media for 14–16 hours, plates were washed twice with PBS, then complete thymidine-free media was added. Eight to ten hours later, 2 mM thymidine was added again for 14–16 hours, cells were washed twice with PBS, then released into thymidine-free media. 50 uM roscovitine was administered for 6 hours following 16 hours nocodazole treatment in A549 cells. Torin1 (50 nM) (Dr. D. Sabatini, MIT) was added for 1 h.

### Biochemistry

**Figure 1:**
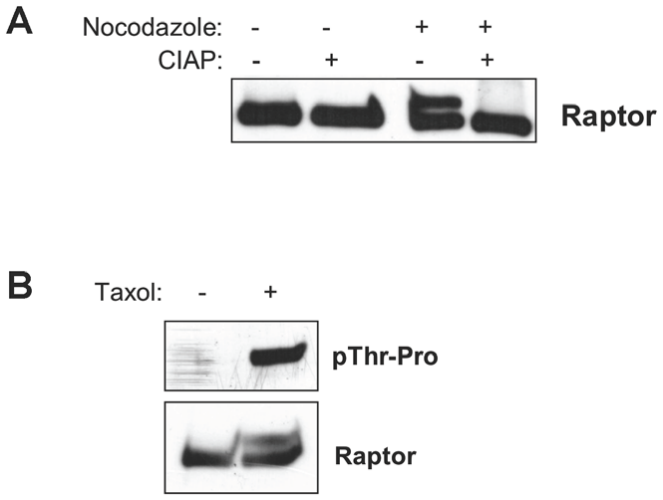
Raptor is phosphorylated on S/T*-P sites in cells treated with nocodazole. (A) Raptor undergoes a mobility shift on SDS-PAGE following nocadazole which is collapsed by phosphatase treatment. Myc-tagged raptor was expressed in HEK293T cells and nocadazole treated for 16 h. Where indicated, immunoprecipitates were treated with or without calf-intestinal alkaline phosphatase (CIP) and then resolved in SDS-PAGE, and subjected to anti-myc immunoblotting. (B) Raptor is recognized by a phospho-threonine proline antibody in mitotic arrested cells. HEK293T cells transiently expressing myc-tagged raptor were treated for 16 h with taxol and immunoprecipitates were immunoblotted with an antibody that recognizes phospho-threonine followed by proline.

**Figure 2:**
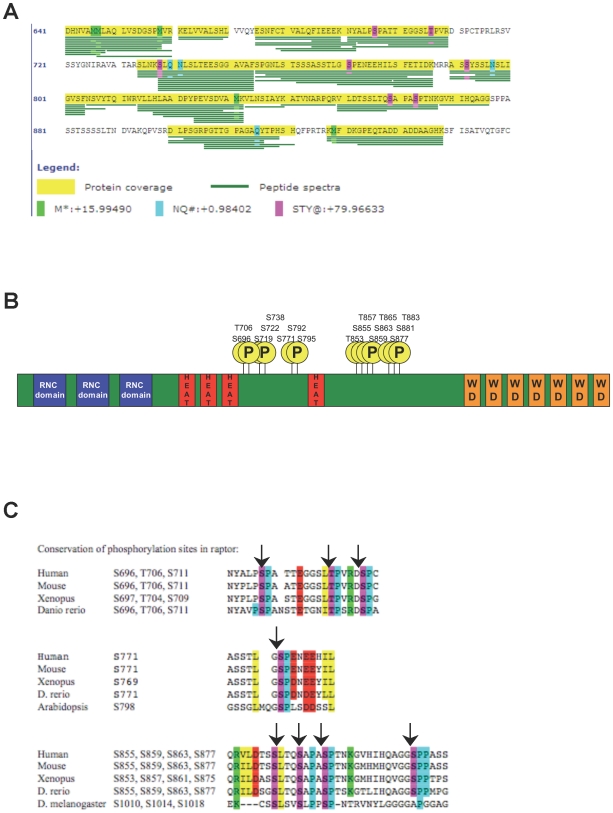
Mass spectrometry analysis of raptor reveals several novel phosphorylation sites. (A) Phosphorylation sites in raptor after nocodazole treatment as detected by LC/MS/MS. The presence of a phosphate moiety is indicated by a magenta colored box. Note that four serine-or threonine sites followed by a proline were detected in this analysis. Sites of oxidation (green) and deamidation (blue) represent in vitro artifacts of the mass spectrometry experiment. (B). Schematic of human raptor domain structure with all known phosphorylation sites found in this and previous studies (for full details see Table S1). Note that most phosphorylation sites cluster in two regions of the protein. (C) Conservation of the indicated phosphorylation sites.

**Figure 3:**
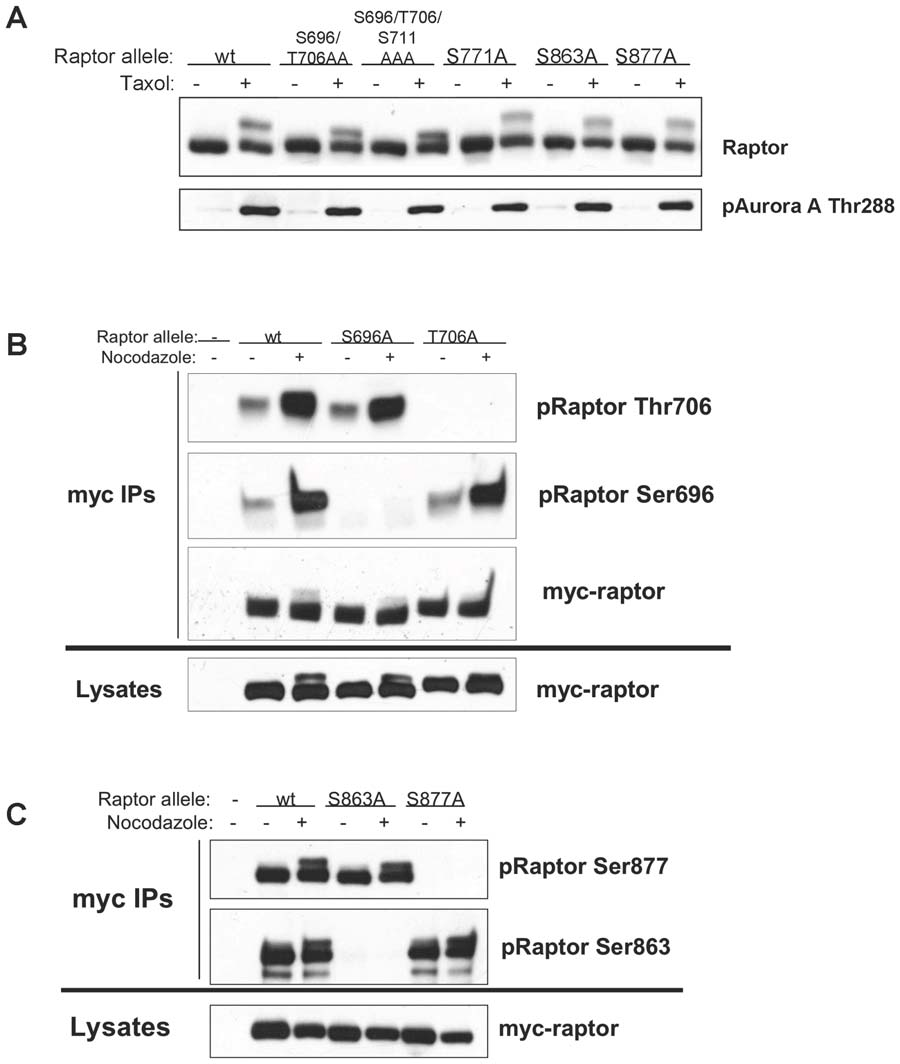
Raptor is phosphorylation on Ser696 and Thr706 during mitosis. (A) The mitotic induced bandshift is collapsed by mutation of Ser696, Thr706, and Thr711. Indicated serine/threonine-to-alanine non-phosphorylatable raptor mutants were expressed in HEK293T cells treated with taxol as in Figure 1. (b, c) Wild-type or non-phosphorylatable raptor alleles were immunoprecipitated from nocadazol treated HEK293T cells and then immunoblotted with indicated site-specific phospho-raptor antibodies. Note specificity of each antisera and that Ser696 and Thr706, but not Ser863 or Ser877 are increased by nocadazole treatment.

**Figure 4:**
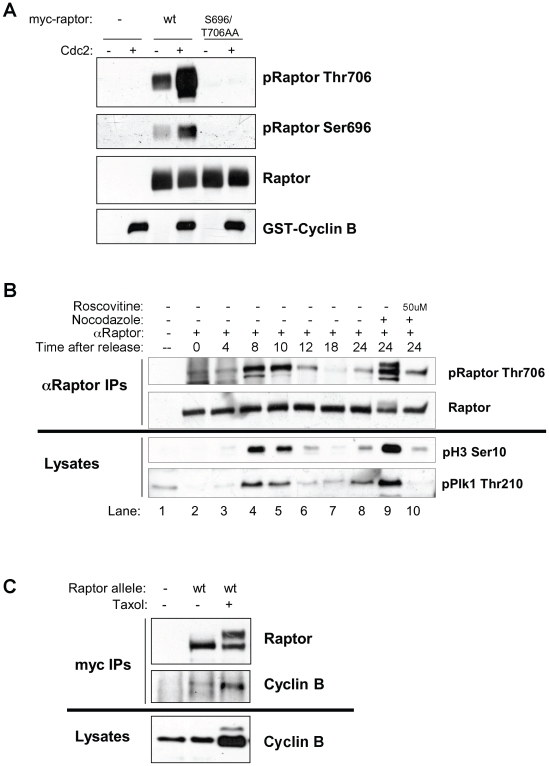
Cdc2 is the raptor Ser696, Thr706 kinase. (A) Purified cdc2 can directly phosphorylate raptor on Thr706 and Ser696 in vitro. Myc-raptor (wild-type or S696/T706AA) was immunoprecipitated from hydroxyurea treated HEK293T cells. Immunoprecipitates were incubated with or without active recombinant Cdc2/cyclin B and immunoblotted with phospho-raptor Ser696, Thr706 or total raptor. (B) Endogenous raptor is phosphorylated on Thr706 in synchronized cells undergoing mitosis, and this phosphorylation is blocked by the CDK inhibitor roscovitine. A549 cells were synchronized by double thymidine block and endogenous raptor was immunoprecipitated at the indicated times after release with an anti-raptor antibody and immunoblotted with phospho-raptor Thr706. Whole cell lysates taken from the same cells were immunoblotted for mitotic markers phospho-histone H3 Ser10 and phospho-Plk1 Thr210. (C) Raptor immunoprecipitates with endogenous cyclin B. Hela cells stably expressing myc-wt raptor with stable knockdown of endogenous raptor treated with or without taxol for 16 hours. myc-tagged raptor was immunoprecipitated and immunoblotted for Cyclin B.

**Figure 5:**
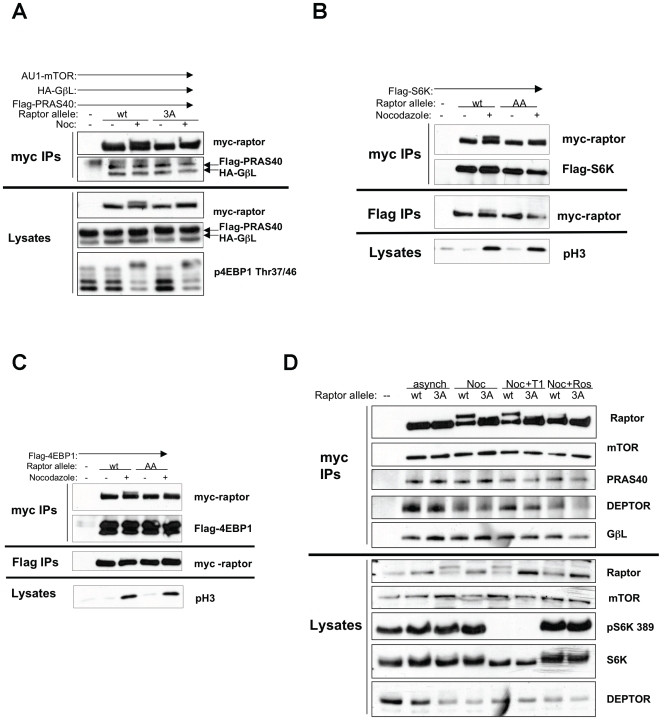
Cdc2 phosphorylation of raptor does not change mTORC1 complexes or signaling. (A) HEK293T cells were transiently transfected with myc-raptor (wild-type or Ser696/Thr706/Ser711AAA: “3A”), AU1-mTOR, HA-GbL and Flag-PRAS40 for 16 hours, followed by 16 hours of nocodazole treatment following addition of fresh media to plates. Cells were lysed and myc-raptor was immunoprecipitated using an antibody against the myc-tag. Immunoprecipitates were resolved by SDS-PAGE and probed with antibodies against the Flag- and HA- tags. (B,C) HEK293T cells were transiently transfected with myc-raptor (wild-type or 3A) and Flag-S6K or Flag-4EBP1. 16 hours later, media was changed and nocodazole was added for 16 hours. Cells were lysed and lysated were split in two; myc-raptor was immunoprecipitated with an antibody against the myc-tag, and Flag-S6K or 4EBP1 were immunoprecipitated with an antibody against the Flag-tag. Immunoprecipitates were resolved by SDS-PAGE and immunoblotted with indicated antibodies. (D) Hela cells stably expressing myc-raptor (wt or 3A) with stable knockdown of endogenous raptor were treated with nocodazole for 16 hours, then Torin1 or roscovitine were added for 4 hours. Cells were lysed and myc-raptor was immunoprecipitated with an antibody against the myc-tag. Immunoprecipitates were resolved by SDS-PAGE and immunoblotted with the indicated antibodies.

For immunoprecipitations, cells were washed with ice cold PBS and collected in lysis buffer 1 (20 mM Tris pH 7.5, 150 mM NaCl, 1% Triton X-100, 50 mM NaF, 1 mM EDTA, 1 mM EGTA, 2.5 mM sodium pyrophosphate, 1 mM b-glycerophosphate, 10 nM Calyculan A, and EDTA-free complete protease inhibitor tablets (Roche) as per manufacturer's directions) for experiments in Figures 1, 2, 3, 4a and 4b or lysis buffer 2 (40 mM HEPES pH 7.5, 150 mM NaCl, 0.3% CHAPS, 50 mM NaF, 1 mM EDTA, 1 mM EGTA, 2.5 mM sodium pyrophosphate, 1 mM b-glycerophosphate, 10 nM Calyculan A and EDTA-free complete protease inhibitor tablets) for Figures 4c and 5. Lysates were incubated on ice for 15 minutes after lysis, then spun at 13,200 rpm at 4°C for 15 minutes. The supernatants were collected and normalized for protein levels by BCA assay (Pierce). Whole cell lysates were incubated with antibodies for 1.5 hours with constant rocking at 4°C, then protein-A or –G sepharose beads (Invitrogen) were added for 1 hour. Immunoprecipitates were washed three times with lysis buffer, and sample buffer was added to 1X final, and samples were boiled at 95°C for 5 minutes. Helas were lysed in boiling SDS-lysis buffer (10 mM Tris pH 7.5, 100 mM NaCl, 1% SDS) and equilibrated by BCA assay. Samples were resolved on 8–12% SDS-PAGE gels, transferred to PVDF and immunoblotted according to the antibody manufacturer's instructions.

### Phosphatase Treatment

Anti-myc immunoprecipitations were performed on cell lysates from HEK293T cells transiently transfected with myc-raptor treated with or without nocodazole. After washing the beads twice in lysis buffer 1, they were washed twice in CIAP buffer (50 mM Tris pH 8.5, 100 uM EDTA) then incubated with 5 uL calf-intestinal alkaline phosphatase (CIAP) (NEB) with constant agitation at 37°C for 30 minutes.

### *In Vitro* Kinase Assays

Anti-myc immunoprecipitations from HEK293T cells transiently transfected with myc-raptor for 16 hours followed by 12 hours of 2 uM hydroxyurea treatment were washed three times with lysis buffer 1, then three times with kinase buffer (25 mM Tris pH 7.5, 10 mM MgCl_2_, 0.1 mM Na_3_VO_4_, 5 mM b-glycerophosphate, 2 mM DTT). Immunoprecipitates were then incubated with 20 uL kinase reaction mix (kinase buffer, 10 uM ATP) with or without 175 ng recombinant Cdc2/cyclin B (Cell Signaling Technologies #7518) at 30° for 30 minutes with constant shaking. Reaction was quenched by addition of sample buffer to 1X and boiling at 95° for 5 minutes.

### LC/MS/MSTandem Mass Spectrometry

For all mass spectrometry (MS) experiments, myc-Raptor immunoprecipitates were separated using SDS-PAGE, the gel was stained with Coomassie blue, and the myc-Raptor band was excised. Samples were subjected to reduction with dithiothreitol, alkylation with iodoacetamide, and in-gel digestion with trypsin or chymotrypsin overnight at pH 8.3, followed by reversed-phase microcapillary/tandem mass spectrometry (LC/MS/MS). LC/MS/MS was performed using an Easy-nLC nanoflow HPLC (Proxeon Biosciences) with a self-packed 75 µm id x 15 cm C_18_ column connected to a LTQ-Orbitrap XL mass spectrometer (Thermo Scientific) in the data-dependent acquisition and positive ion mode at 300 nL/min. MS/MS spectra collected via collision induced dissociation in the ion trap were searched against the concatenated target and decoy (reversed) single entry Raptor and full Swiss-Prot protein databases using Sequest (Proteomics Browser Software, Thermo Scientific) with differential modifications for Ser/Thr/Tyr phosphorylation (+79.97) and the sample processing artifacts Met oxidation (+15.99), deamidation of Asn and Gln (+0.984) and Cys alkylation (+57.02). Phosphorylated and unphosphorylated peptide sequences were identified if they initially passed the following Sequest scoring thresholds against the target database: 1+ ions, Xcorr ≥2.0 Sf ≥0.4, P≥5; 2+ ions, Xcorr ≥2.0, Sf ≥0.4, P≥5; 3+ ions, Xcorr ≥2.60, Sf ≥0.4, P≥5 against the target protein database. Passing MS/MS spectra were manually inspected to be sure that all **b-** and **y-** fragment ions aligned with the assigned sequence and modification sites. Determination of the exact sites of phosphorylation was aided using FuzzyIons and GraphMod and phosphorylation site maps were created using ProteinReport software (Proteomics Browser Software suite, Thermo Scientific). False discovery rates (FDR) of peptide hits (phosphorylated and unphosphorylated) were estimated below 1.5% based on reversed database hits.

## Results

### Raptor Is Phosphorylated on S/T-P Sites in Cells Stalled in Mitosis with Nocodazole

We have shown previously that cells undergoing energy stress arrest in G2/M and that if they lack the ability to downregulate mTORC1 signaling during energy stress, they proceed inappropriately into mitosis [10]. In the course of further examining how mTORC1 signaling is regulated during mitosis, we observed that stalling cells in mitosis through use of the microtubule destabilizing drug nocodazole caused a shift in the mobility of raptor on SDS-PAGE, and this was reversed by in vitro phosphatase treatment of the immunoprecipitates (Figure 1a). Similar results were also seen with the microtubule stabilizing drug taxol, which also promotes mitotic arrest (Figure 1b). Phosphorylation-induced mobility shifts are often indicative of phosphorylation on serine/threonine-proline residues, and indeed we see an increase in immunoreactivity of an anti-phospho-threonine-proline antibody on raptor immunoprecipitates isolated from arrested cells compared to asynchronous cells (Figure 1b).

### Mass Spectrometry Analysis of Raptor Reveals Several Novel *In Vivo* Phosphorylation Sites

In attempts to identify the residues of raptor responsible for the nocodazole-induced bandshift, microcapillary liquid-chromatography/tandem mass spectrometry (LC/MS/MS) was performed on raptor immunoprecipitated from cells with or without nocodazole treatment. We identified several novel phosphorylation sites from this analysis, including Ser696, Thr706, Ser738, Ser771 and Ser877 (Figure 2a, Table S1). Of these sites, Ser696, Thr706, Ser771, and Ser863, Ser877 are S*-P sites, all of which are evolutionarily conserved through vertebrates (Figure 2b). Interestingly, mapping our phosphorylation sites along with all those from phospho-proteomic databases including PhosphoSitePlus (www.phosphosite.org; [12]) reveals that phosphorylation sites within raptor cluster to two regions located between the HEAT repeat region and the WD-repeat containing C-terminal of raptor (Figure 2c).

### Raptor Is Phosphorylated on Ser696 and T706 during Mitosis

Next we examined whether mutation of any of the identified serine/threonine-proline sites to alanine would alter the mobility shift induced in raptor upon nocodazole or taxol treatment. From this analysis, we discovered that an allele of raptor with mutation of Ser696 and Thr706 to alanine, showed reduced band-shifting (Figure 3a). Mutation of the adjacent serine-proline residue, Ser711, to alanine in combination with 696/706 was found to further collapse the bandshift, suggesting that S711 is also phosphorylated in cell blocked in mitosis. Mutation of other reported S/T*-P sites in raptor did not collapse the bandshift (Fig. 3a, data not shown).

To directly determine the residues of raptor up-regulated following nocodazole treatment, phospho-specific-antibodies against Ser696, Thr706, Ser877, and Ser863 were generated and verified as recognizing only wild-type myc-tagged raptor immunoprecipitated from HEK293T cells, but not raptor mutated at the specified phospho-acceptor residue (Figure 3b, c). Treatment with nocodazole to block cells in mitosis greatly increased phosphorylation of raptor on Ser696 and Thr706 (Figure 3b) but notably, not any of the other S/T*-P sites tested (Figure 3c).

### Cdc2 Is the Raptor Ser696, Thr706 Kinase

Having identified several residues of raptor phosphorylated in cells blocked in mitosis, we next sought to identify the upstream kinase for Ser696 and Thr706. Knowing the upstream kinase was active following nocodazole treatment, and was proline-directed, we decided to examine the mitotic CDK family member Cdc2. First we tested whether recombinant Cdc2-cyclin B kinase complexes were capable of in vitro phosphorylation of raptor immunoprecipitated from hydroxyurea treated HEK293T cells (and hence derived from cells where Cdc2 would be inactive) (Figure 4a). Indeed cdc2/cyclin B induced robust phosphorylation of raptor in vitro on Thr706 and Ser696.

To examine whether Phospho-Thr706 can be detected during natural mitotic progression and is not simply due to kinases activated by microtubule stress, we synchronized A549 cells using double thymidine block and endogenous raptor was immunoprecipitated at various timepoints following thymidine release and immunoblotted with the phospho-raptor Thr706 antibody (Figure 4b). Mitotic entry peaked at 8 to 10 hours following thymidine release in these cells as demarcated by increased mitotic markers phospho-histone H3 and phospho-Plk1, coinciding exactly with maximal Thr706 phosphorylation on endogenous raptor (Fig 4b). Importantly, endogenous raptor phosphorylation was observed during mitosis in the synchronized cells similar to that observed following nocadozole treatment when all of the cells are arrested in mitosis. We further examined cdc2 involvement through acute treatment of nocodazole arrested cells with the cdc2 inhibitor roscovitine. Roscovitine resulted in inhibition of endogenous phospho-raptor Thr706 (Fig. 4b, lanes 9–10).

Finally, we examined whether we could detect and in vivo association between raptor and the cdc2/cyclin B kinase complex. Utilizing Hela cells stably expressing low levels of tagged raptor, raptor immunoprecipitates from cycling or taxol-arrested cells revealed the presence of endogenous cyclin B (Fig 4c).

### Cdc2 Phosphorylation of Raptor on Ser696, Thr706 Does Not Impact Mtorc2 Complex Formation

mTORC1 is a multi-protein complex whose function requires proper association of all components [13]. To test whether Cdc2 phosphorylation of raptor might change the association of various components of mTORC1, epitope tagged cDNAs of the components of mTORC1 were expressed in HEK293E cells with or without nocodazole. No changes in the amount of HA-GbL or Flag-PRAS40 that co-immunoprecipitated with myc-tagged raptor were observed with either nocodazole treatment or raptor allele (Figure 5a). However, in whole cell lysates taken from the same cells, endogenous 4EBP1 phosphorylation increased, which occurs regardless of raptor allele.

It has been demonstrated that raptor acts as a scaffolding protein between mTOR and its substrates [14], [15], so we tested whether Cdc2 phosphorylation of raptor changes the association of known mTOR substrates S6K and 4EBP1. We observed no significant change in the amount of Flag-tagged S6K or 4EBP1 immunoprecipitated with myc-raptor in HEK293T cells treated with or without nocodazole regardless of raptor allele (Figure 5 b, c), and the same is true of the reciprocal immunoprecipitations.

To confirm these results in a more physiological system, cell lines stably over-expressing a low level of myc-tagged raptor alleles with stable knockdown of endogenous raptor with short hairpin RNA that targets the 3′ UTR of raptor [16] were generated in Hela cells. No changes in the ability of endogenous mTORC1 components to co-immunoprecipitate with myc-raptor were observed with either nocodazole treatment or different raptor alleles (Figure 5d). Taken together, these data suggest that phosphorylation of raptor by Cdc2 does not significantly change the composition of mTORC1 nor the ability of mTOR to signal to downstream substrates S6K or 4EBP1 during mitosis.

## Discussion

How signaling pathways coupled to nutrient uptake and expenditure couple to the cell cycle machinery and proliferation control has been an area of increasing investigation. The mTORC1 signaling pathway is a critical integrator of environmental inputs into protein translation and cell growth. However, the precise role of mTORC1 signaling in mitotic progression remains enigmatic [17].

Our previous studies indicated the presence of G2/M metabolic checkpoint enforced by AMPK in a manner dependent on its ability to phosphorylate raptor and suppress mTORC1 activity. Cells expressing non-phosphorylatable alleles of raptor continued to progress through mitosis unabated unlike those expressing wild-type raptor, and ultimately displayed increased rates of apoptosis. Consistent with an AMPK/mTORC1 dependent checkpoint, AMPKα2 and its upstream kinase LKB1 were isolated in an RNAi screen for modulators of G2/M in mammalian cells [18]. Furthermore, increased phosphorylation at the AMPKα2 activation loop was observed in a proteomic study for kinases activated during G2/M [19] and more recently, activated AMPK has been proposed to reside at the mitotic spindle [20], hinting at both spatial and temporal regulation of AMPK in mitosis which may restrict or target its regulation of mTOR to specific locations or phases of mitosis.

Previous studies also suggest that mTOR signaling plays a positive role in the progression through mitosis in a variety of species. In budding yeast, a temperature sensitive allele of raptor or rapamycin-treatment of cells both induce mitotic delay and a prolonged G2 [21]. In contrast, in fission yeast rapamycin induces early mitotic onset in synchronized cultures [22], though in both yeasts, TOR activity has been tied to the control of Polo kinase activation. Further work is needed in each of these biological settings to further dissect the role of TOR in mitotic control.

We demonstrate here that the mitotic kinase cdc2 directly phosphorylates raptor during mitosis, though we have been unable to demonstrate the contribution those phosphorylations play to overall mitotic progression or mTORC1 signaling during mitosis in the tumor cell settings we have examined thus far. Importantly, our data suggests there may be additional cdc2 sites beyond Ser696, Thr706, and Ser711 in raptor and until these sites are fully identified, the phenotype of a fully cdc2 non-phosphorylatable raptor remains unknown. Nontheless, the cdc2 sites in raptor may be more critical for growth control in non-tumorigenic settings, which is an area requiring further investigation.

One additional complicating factor is these analyses is the fact that cdc2 has been reported to directly phosphorylate both S6K1 [23], [24], [25] and 4ebp1 directly [26], [27]. Indeed the well-characterized Ser65 and Ser70 phosphorylation sites in 4e-bp1 have been proposed to be sites of phosphorylation by cdc2, events that are dependent on mTORC1 activity. Additionally, cdc2-dependent phosphorylation of EE2K was shown to be suppressed by amino acid deprivation and increased in cells lacking TSC2, conditions that respectively serve to inhibit and stimulate mTORC1 signaling, leading to the suggesting that mTORC1 activity may serve to contribute to cdc2-dependent regulation of EEF2K [28]. The possibility exists therefore that both cdc2 and mTORC1 kinase complexes serve to inter-regulate one another depending on the precise timing and localization of each during different stages of mitosis. The fact that several components of the mTORC1 pathway are targeted by cdc2 may result in no single one of them being critical in isolation as a cdc2 target whose phosphorylation is absolutely required for mitotic progression.

A complication of much of the previous literature studying the effect of mTOR on G2/M progression utilizing rapamycin is that recent finding from several labs that rapamycin does not fully inhibit mTORC1 kinase activity. Kinase inhibitors directed at mTOR itself yield changes in mTORC1 signaling and growth arrest phenotypes more similar to RNAi for raptor [29], [30], [31]. Importantly, these effect of mTOR kinase inhibitors were demonstrated to be independent of the mTORC2 complex and its function [29], [30]. These findings are also consistent with a variability of rapamycin in inhibiting S6K1 signaling but not 4ebp1 phosphorylation universally in mammalian cells, unlike RNAi or genetic deletion of raptor or mTOR [30], [32]. The inability of rapamycin to suppress 4ebp1 phosphorylation indicates that previous studies in mammalian cells studying effects of rapamycin on mitosis were not accounting for the full role of mTORC1. Future studies using these new direct mTOR kinase inhibitors will be needed to fully dissect its requirement in different stages of mitotic progression.

Additional tools including phospho-specific antibodies which can work for immunolocalization may better reveal where the population of cdc2-phosphorylated raptor and 4ebp1 are during the different stages of mitosis. Understanding how AMPK activity and mTOR activity are controlled spatially and temporally during mitosis will undoubtedly lead to fundamental insights into how nutrients control cell division as well as to how protein translation is coupled to timely cell cycle exit during differentiation or stem cell renewal. A deeper understanding of how mTORC1 controls cell cycle progression is essential for use of targeted mTOR inhibitors in the treatment of cancer and many other mTOR-related pathologies [33].

## Supporting Information

Table S1 All identified in vivo phosphorylation sites in raptor. Conservation is indicated and predicted kinases from Scansite are listed in black. Reported in vivo kinases are indicated in red.
(0.76 MB TIF)
