# Supplementary figures and images for: Raptor is Phosphorylated by cdc2 during Mitosis
Source: PLoS One. 2010 Feb 12;5(2):e9197. doi: 10.1371/journal.pone.0009197 (PMC2820552; doi:10.1371/journal.pone.0009197)

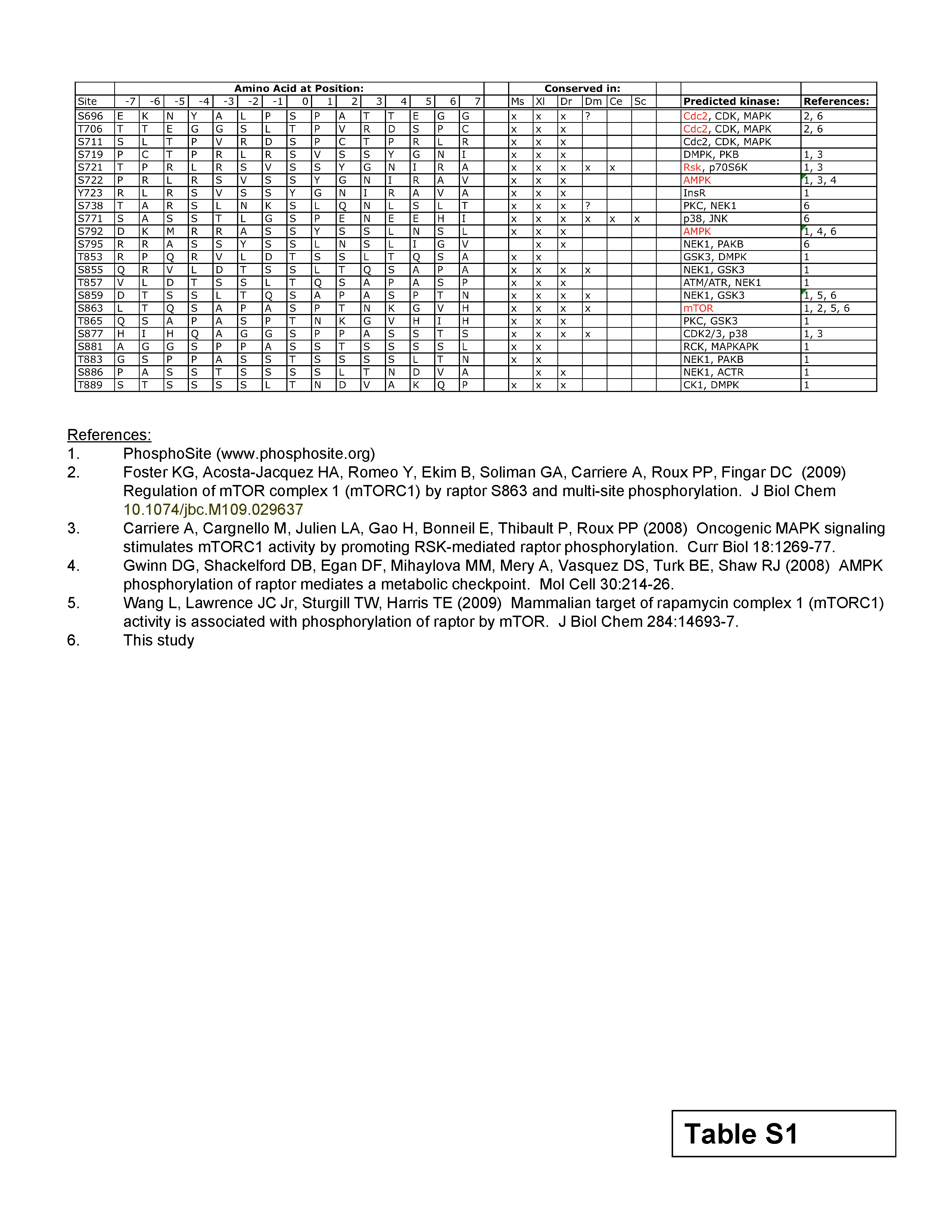

Supplement: Table S1 — All identified in vivo phosphorylation sites in raptor. Conservation is indicated and predicted kinases from Scansite are listed in black. Reported in vivo kinases are indicated in red. (0.76 MB TIF) [file pone.0009197.s001.tif]
